# High susceptibility of wild *Anopheles funestus* to infection with natural *Plasmodium falciparum* gametocytes using membrane feeding assays

**DOI:** 10.1186/s13071-016-1626-y

**Published:** 2016-06-14

**Authors:** Cyrille Ndo, Edmond Kopya, Benjamin Menze-Djantio, Jean Claude Toto, Parfait Awono-Ambene, Gareth Lycett, Charles S. Wondji

**Affiliations:** Malaria Research Laboratory, Organisation de Coordination pour la lutte contre les Endémies en Afrique Centrale (OCEAC), P.O. Box 288, Yaoundé, Cameroon; Department of Vector Biology, Liverpool School of Tropical Medicine, Pembroke Place, Liverpool, L3 5QA UK; Faculty of Medicine and Pharmaceutical Sciences, University of Douala, P.O. Box 2701, Douala, Cameroon; Faculty of Sciences, University of Yaoundé I, P.O. Box 337, Yaoundé, Cameroon; Research Unit Liverpool School of Tropical Medicine, OCEAC, P.O. Box 288, Yaoundé, Cameroon

**Keywords:** Experimental infection, *Anopheles funestus*, *Plasmodium falciparum*, Membrane feeding

## Abstract

**Background:**

*Anopheles funestus* is a major vector of malaria in sub-Saharan Africa. However, because it is difficult to colonize, research on this mosquito species has lagged behind other vectors, particularly the understanding of its susceptibility and interactions with the *Plasmodium* parasite. The present study reports one of the first experimental infections of progeny from wild-caught *An. funestus* with the *P. falciparum* parasite providing a realistic avenue for the characterisation of immune responses associated with this infection.

**Methods:**

Wild-fed resting *An. funestus* females were collected using electric aspirators and kept in cages for four days until they were fully gravid and ready to oviposit. The resulting eggs were reared to adults F1 mosquitoes under insectary conditions. Three to five day-old *An. funestus* F1 females were fed with infected blood taken from gametocyte carriers using an artificial glass-parafilm feeding system. Feeding rate was recorded and fed mosquitoes were dissected at day 7 to count oocysts in midguts. Parallel experiments were performed with the known *Plasmodium*-susceptible *An. coluzzii* Ngousso laboratory strain, to monitor our blood handling procedures and infectivity of gametocytes.

**Results:**

The results revealed that *An. funestus* displays high and similar level of susceptibility to *Plasmodium* infection compared to *An. coluzzii*, and suggest that our methodology produces robust feeding and infection rates in wild *An. funestus* progeny. The prevalence of infection in *An. funestus* mosquitoes was 38.52 % (range 6.25–100 %) and the median oocyst number was 12.5 (range 1–139). In parallel, the prevalence in *An. coluzzii* was 39.92 % (range 6.85–97.5 %), while the median oocyst number was 32.1 (range 1–351).

**Conclusions:**

Overall, our observations are in line with the fact that both species are readily infected with *P. falciparum*, the most common and dangerous malaria parasite in sub-Saharan Africa, and since *An. funestus* is widespread throughout Africa, malaria vector control research and implementation needs to seriously address this vector species too. Additionally, the present work indicates that it is feasible to generate large number of wild F1 infected *An. funestus* mosquitoes using membrane feeding assays, which can be used for comprehensive study of interactions with the *Plasmodium* parasite.

**Electronic supplementary material:**

The online version of this article (doi:10.1186/s13071-016-1626-y) contains supplementary material, which is available to authorized users.

## Background

Despite recent progress in reducing malaria burden, it remains one of the most debilitating diseases in the tropical world with 198 million cases reported in 2013, leading to more than 500,000 deaths [1]. In sub-Saharan Africa, where about 90 % of all malaria deaths occur, mosquitoes from *Anopheles gambiae* complex and *Anopheles funestus* group are the most efficient vectors [2–5], while *P. falciparum* is the most common and dangerous parasite responsible for most cases of severe malaria [1].

Current efforts to control or to eliminate malaria rely heavily on vector control interventions such as large-scale distribution of long-lasting insecticide-treated nets (LLINs) and in some places through indoor residual house spraying (IRS) [6–8]. In parallel, research on new and innovative malaria control tools that could enhance the efficacy or complement existing approaches is needed. One of the approaches being pursued is the genetic replacement of vector populations with non-vectors in order to disrupt parasite transmission [9–11]. However, this requires first of all a good understanding of the complex and specific interactions between the *Plasmodium* parasites and its *Anopheles* vectors, notably the associated immune response.

Obtaining sufficient *Plasmodium* experimentally infected mosquitoes is the first and one of the most challenging steps of research on *Anopheles-Plasmodium* interactions. In *An. gambiae* which is easier to colonize and adapts well to feed on the artificial parafilm membrane, successful experimental infections are commonly performed using either the rodent *P. berghei* or cultured or recently field collected human *P. falciparum* parasites. This has allowed the identification of important mosquito factors that may prove to be favourable targets for novel interventions towards blocking malaria transmission [12–14].

If such progress has been achieved in *An. gambiae*, little advance has been observed for the other major malaria vector *An. funestus*. It is known that *An. funestus* exhibits important differences to *An. gambiae* in term of its biology, genetics and ecology as shown by the difficulty of rearing and feeding this species in laboratory conditions [3]. Such difficulty has largely slowed the pace of research on this species, particularly its interactions with the *Plasmodium* parasite.

By collecting indoor resting gravid females and force them to lay eggs, it is now possible to generate large F1 colonies of *An. funestus* for controlled experimentation [15, 16]. This method already allowed study of insecticide resistance mechanisms throughout Africa [17–21]. Conversely, only two studies have investigated *An. funestus* susceptibility to the rodent parasite *P. berghei* [22, 23], and none have been performed with the natural human parasite *P. falciparum*. Despite similarities between these two *Plasmodium* species, important differences in interactions with mosquito vectors have been reported [14, 24]. This observation point out the necessity of following up discoveries in laboratory model systems with studies on natural parasite-mosquito interactions, particularly in the perspective of investigating molecular bases of *An. funestus* immune response to *Plasmodium* infection.

This paper reports the first experiments in which first generation of wild-caught *An. funestus* mosquitoes are infected with natural *P. falciparum* gametocytes using an artificial blood feeding system.

## Methods

### Mosquito collections

*Anopheles funestus* mosquitoes were collected in Mebelong (6°46′N, 11°70′E), a village situated in humid savannah region, about 350 km North of Yaoundé, the capital city of Cameroon. This site is characterised by a high density of mosquitoes resting indoors.

Resting mosquitoes were collected using electric aspirators (Rule In-Line Blowers, Model 240). In the field, *An. funestus* mosquitoes were sorted from other *Anopheles* species based on morphological criteria [25, 26] and kept in paper cups for four days or more until they were fully gravid and ready to lay eggs. Once brought back in the insectary, females were allowed to oviposit individually using a forced egg-laying method [15]. After oviposition, all the carcasses were preserved in individual tubes containing desiccant for molecular identification [27, 28] and for enzyme-linked immunosorbent assay (ELISA), to assess *Plasmodium* infection rate. All other *Anopheles* species collected as well as *An. funestus* specimens that died before reaching the insectary were also preserved for ELISA, to identify blood meal source and assess *Plasmodium* infection rate according to [29, 30] and [31], respectively. Positive samples to ELISA were determined by reading the optical densities at 405 nm using an ELISA plate reader (BioTek ELx800, Swindon, UK).

We used the *An. coluzzii* Ngousso laboratory strain as a control sample to monitor for the effectiveness of blood handling procedure and infectivity of gametocytes during infection experiments. The Ngousso strain, routinely maintained at OCEAC, has been extensively used in experimental infection studies and is known to be well adapted to feed through artificial parafilm membrane and is highly susceptible to *Plasmodium* infection [32]. *Anopheles funestus* and *An. coluzzii* females were fed with blood from same gametocyte carriers to directly compare prevalence of infection.

### Identification of gametocyte carriers

Parasitological surveys to detect gametocyte carriers were conducted during a high transmission period from March to June 2015. Children aged between 5 and 11 were recruited at local schools of the locality of Okola (4°01′N, 11°22′E), a rural area situated about 25 km from Yaoundé. Blood samples from each child enrolled in the study were screened by direct microscopic (100×) visualization of *Plasmodium* parasites on thick blood smears stained with 10 % Giemsa. Trophozoite density was determined using semi quantitative count (thick film) method following a semi-quantitative scale [33]. Gametocyte density was estimated by microscopy read against 500 leucocytes, assuming a standard white blood cell concentration of 8,000/μl [34].

### Experimental infections

Gametocyte carriers were selected based on parasite density in blood after their haemoglobin content was first measured using Haemocue (HaemoCue^®^ AB, Angelholm, Sweden). Children with abnormally low haemoglobin rate were treated for anaemia and their blood was not used. For selected children, a volume of 3–4 ml of blood was taken by venepuncture into heparinized tubes. The blood was immediately centrifuged at 37°C and the serum was replaced by the same volume of European AB serum (Sigma-Aldrich, Taufkirchen, Germany), pre-warmed at the same temperature. The reconstituted blood was offered to mosquitoes immediately.

Groups of 80 to 100, 3–5-day-old F1 female mosquitoes were counted into paper cups covered with mosquito netting and starved 12h before experiments. 450 μl aliquots of reconstituted blood were placed in each glass feeder with a parafilm membrane maintained at 37°C using a circulating heating water bath (Fisher Scientific INC, Isotemp 4500H5P, Pittsburgh USA). Mosquitoes were allowed to feed for 45min, after which unfed and partially fed mosquitoes were removed. Mosquitoes were maintained in separate cups in the insectary at 26 ± 2°C and 70 to 80 % relative humidity, with a constant supply of 10 % sucrose and daily mortality was recorded. Part of fed mosquitoes were preserved in RNAlater for future transcriptomic analysis. The remaining ones were dissected at day 7 post-infection in a drop of 0.4 % mercurochrome and stained midguts were examined for detection and quantification of oocysts under light microscopy (40×) [35].

### Data analysis

Parameters recorded included number of mosquitoes fed per batch, mortality to day 7, number of mosquitoes infected at oocyst stage and number of oocysts per midgut. For each experiment, the median oocyst number was determined and mean oocyst number per midgut was calculated by dividing the total number of oocysts counted by the number of mosquitoes found infected after dissection.

Fisher’s exact test was used to compare prevalence of infection between species. The median oocyst numbers were compared using the nonparametric Mann–Whitney test. Correlation between the prevalence of infection, the median number of oocyst and the gametocytemia was analysed using Spearman’s correlation. All statistical analyses were performed using Graph Pad prism V.5 and *P*-values of 0.05 or less were considered as significant.

## Results

### Mosquito population and rearing

During eight days, a total of 1,918 mosquitoes belonging to three genera were collected in Mebelong. *Anopheles* mosquitoes were by far the most abundant representing 99.48 % of mosquitoes, while *Culex* and *Mansonia* accounted for less than 1 %. Three *Anopheles* species were identified in the locality: *An. funestus* (*s.l*.) the main vector species (*n* = 1,831; 95.96 %), *An. gambiae* (*s.l*.) (*n* = 74; 3.88 %) and *An. pharoensis* (*n* = 3; 0.16 %). According to the molecular identification of a subsample of 890 mosquitoes, *An. funestus* (*s.s*.) (99.43 %) and *An. leesoni* (0.57 %) were the two members of the *An. funestus* group present in the locality.

*Anopheles funestus* (*s.s*.) (hereafter *An. funestus*) survival rate was high with 73.73 % (*n* = 1,350) of mosquitoes reaching the insectary alive (after a 350 km journey in cooling box) and used for egg production. Similarly, high oviposition (80.81 %) and egg hatch (85.43 %) rates were recorded, indicating that large majority of mosquitoes were already inseminated at the time they were collected.

Preliminary ELISA analysis revealed that *An. funestus* actively transmits malaria in Mebelong, with anthropophilic and infection rates of 100 % (*n* = 100) and 3.7 % (*n* = 656), respectively.

### Parasitological survey

In total 1,091 children aged between five and 11 years were examined and the prevalence of detected asexual malaria was 40 % in the survey population. Among the infected children, the result of blood smears ranged from 1+ to 4+, with the majority of them failing in the 1+ (53.35 %) and 2+ (35.10 %) groups [34] (Table 1). According to this prevalence rate, our survey area could be classified as mesoendemic for malaria, although few children above ten years were included in the study [36, 37]. The prevalence of gametocyte carriers identified by blood smear in the total survey population examined was approximately 10 %.

**Table 1:** Summary of parasitological surveys. Data in parentheses represent prevalences. Gametocyte carriers were detected among children aged between 5 and 11 after screening of their blood samples by direct microscopic visualisation of *Plasmodium* parasites on thick blood smears stained with 10 % Giemsa. *Plasmodium* trophozoite density was determined using semi quantitative count (thick film) method

School	Number	TPF+	TPF++	TPF+++	TPF++++	Total tropozote	Gametocyte
	examined					carriers	carriers
Nkolyada	74	22	15	1	0	38 (51.35)	4 (5.41)
Elig-Onana	102	30	23	13	0	66 (64.71)	9 (8.82)
Okola G2	111	25	11	5	0	41 (36.94)	7 (6.31)
Zamengoé	210	58	22	11	1	92 (43.81)	24 (11.43)
Nkolngock	110	25	22	2	0	49 (44.54)	12 (10.91)
Ndangueng	105	18	9	5	3	35 (33.33)	8 (7.62)
Mvoua	140	28	27	4	1	60 (42.86)	23 (16.43)
Levalombédé	239	25	23	3	1	52 (21.76)	18 (7.53)
Total	1,091	231	152	44	6	433 (39.69)	101 (9.26)

TPF levels: +, 1–10 trophozoites/100 microscopic thick film fields; ++, 1–10 trophozoites/10 microscopic thick film fields; +++, 1–10 trophozoites/single microscopic thick film field; ++++, > 10 trophozoites/single microscopic thick film field

*Abbreviation:* *TPF* trophozoite of *P. falciparum*

### *Plasmodium falciparum* infection in *An. funestus*

Fourteen infection experiments were carried out using blood from different gametocyte carriers. In total, 9,728 *An. funestus* females were given access to infected blood through an artificial parafilm membrane and 2,518 successfully fed, corresponding to a global feeding rate of 26 % (Table 2). However, feeding rate significantly varied across experiments ranging from 18 to 47 %. Monitoring of the mortality to day 7 for nine batches of infected mosquitoes revealed a mortality rate between 0 and 69.23 % (mean 38.05 %).

**Table 2:** Summary of experimental infection parameters in *An. funestus*. Mosquitoes aged between 3 to 5 days were given infected blood through artificial parafilm membrane and midgut were dissected at day 7 post-infection for oocyst detection under light microscopy. Feeding rate was calculated by dividing the number of mosquitoes alive in cups after blood feeding by the number successfully fed. Prevalence of infection was calculated by dividing the number of mosquitoes infected at oocyst stage by the total number of mosquito dissected

Experiment	Gametocyte density	Feeding rate (%)	Dissected	Infected	Prevalence of infection (%)	Total oocyst count	Oocyst range (Min-Max)	Median oocyst load
N°1	–	24.52	32	11	34.37	45	1–15	2.5
N°2	–	19.10	36	22	61.11	162	1–18	7
N°3	–	28.89	21	0	0	0	–	–
N°4	–	25.25	32	2	6.25	4	1–3	1
N°5	–	23.78	39	6	15.38	9	1–4	1
N°6	–	46.53	24	3	12.50	4	1–2	1
N°7	–	26.03	84	0	0	0	–	–
N°8	–	33.80	30	0	0	0	–	–
N°9	96	31.38	59	42	71.19	241	1–16	8
N°10	32	19.19	43	14	32.56	20	1–2	1
N°11	368	18.81	52	17	32.69	40	1–6	2.5
N°12	880	18.37	9	9	100	539	1–139	60
N°13	80	31.92	105	37	35.24	96	1–8	4
N°14	16	27.61	81	3	3.7	5	1–2	1
All	–	25.89	647	166	25.66	1165	1–139	12.5

*Plasmodium falciparum* gametocytes were infective for mosquitoes in 11 (78.57 %) of 14 experiments with prevalence of infection ranging from 3.7 to 100 % (mean 25.70 %). A total of 1,165 oocysts were counted in all infected midguts and the global median oocyst number was 12.5 (mean 7.02) (Table 2). Taking each experiment individually, the number of oocysts observed in a midgut ranged from 0 to 139 and median oocyst number varied from 1 to 60 (range for means 1.43–59.88).

**Fig. 1:**
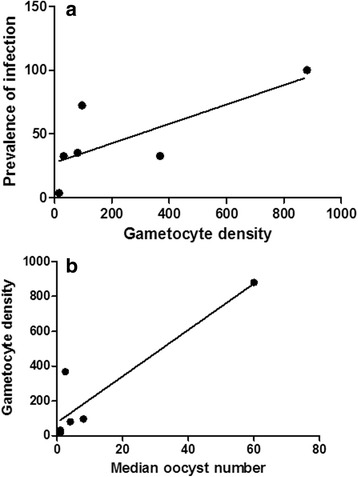
Infection parameters of wild F1 *An. funestus* progeny*.* **a** Linear regression and correlation between parasite density in blood and prevalence of infection. **b** Linear regression and correlation between parasite density in blood and median oocyst number in midguts

The gametocyte density was determined for 6 successful experiments and ranged from 16 to 880 gametocytes/μl of blood. There was a general trend showing that prevalence of infection and oocyst number in midguts increased with gametocyte density in blood samples (Fig. 1). However, the correlation was statistically significant only for oocyst number (*r*_*s*_ = 0.849, *P* = 0.009) and prevalence of infection (*r*_*s*_ = 0.557, *P* = 0.088).

### Comparative analysis of the susceptibility of *An. funestus* and *An. coluzzii* to *P. falciparum* infection

The same infected blood was given to *An. funestus* and *An. coluzzii* mosquitoes simultaneously in 12 parallel experiments for comparison. The feeding rate was two to three-fold higher in *An. coluzzii* lab strain compared to the wild *An. funestus* (Additional file 1: Table S1). Among the 11 experiments in which more than five mosquitoes were dissected for both species, ten (90.91 %) resulted in at least one infected mosquito in *An. funestus* compared to nine (81.82 %) in *An. coluzzii*.

**Fig. 2:**
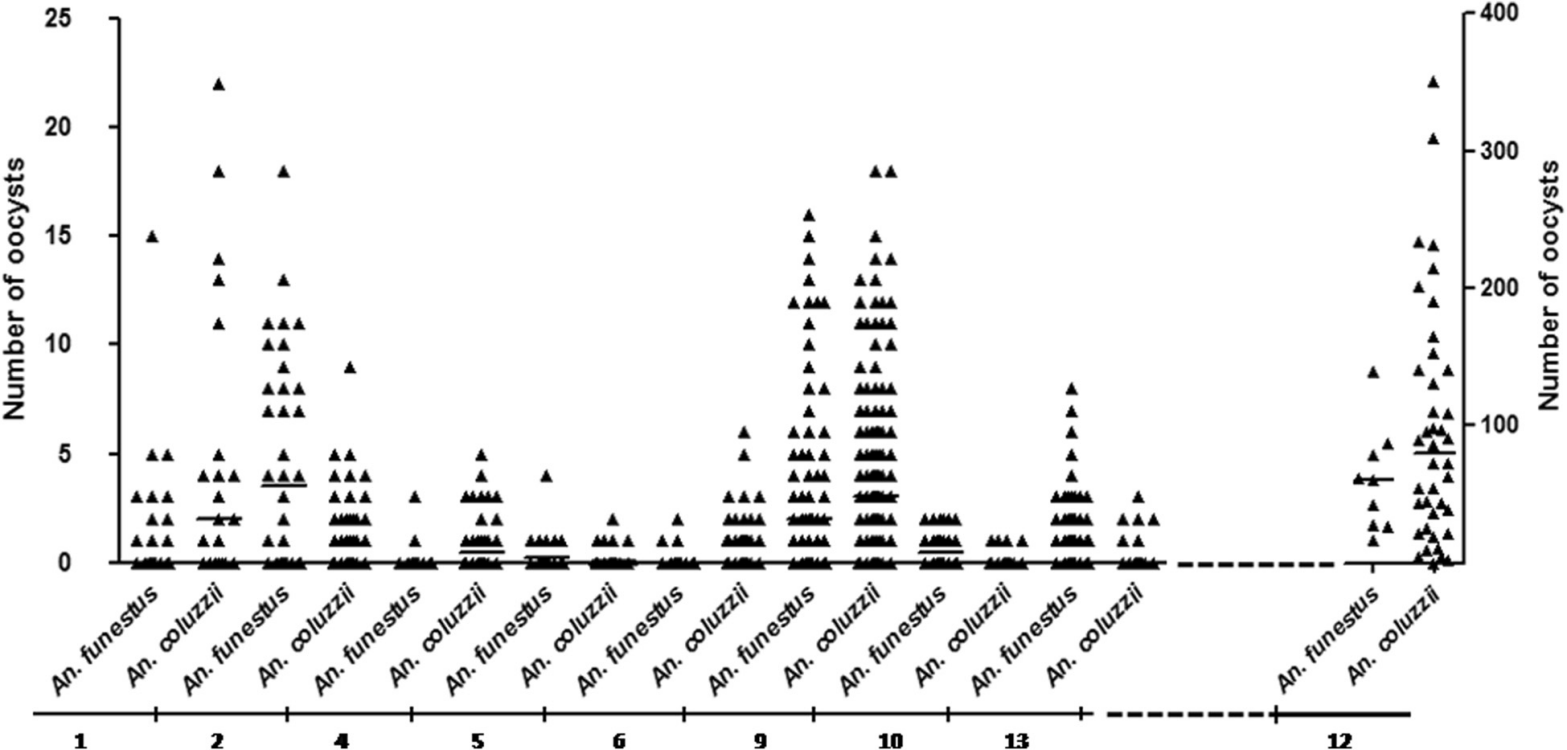
Variation of number of oocyst in individual *An. funestus* and *An. coluzzii* midguts in nine experiments. Each triangle represents the number of oocysts in an individual midgut. Dotted lines on the X-axis separate experiment 12 from the rest to better see variation in oocyst number in all experiments. The Y-axis on the left is related to experiments 1, 2, 4, 5, 6, 9, 10 and 13. The Y-axis on the right is only related to experiment 12, for which very high number of oocysts in individual midgut was recorded

Overall, the two species showed high and comparable susceptibility to *P. falciparum* infection. Based on the nine experiments which were successful in both species, the prevalence of infection in *An. funestus* was 38.52 % (range 6.25–100 %) and the median oocyst number was 12.5 (mean 7.68; range 1–139). In parallel, the prevalence of infection in *An. coluzzii* was 39.92 % (range 6.85–97.5 %), while the median oocyst number was 32.1 (mean 20.33; range 1–351) (Fig. 2). These two parameters did not vary significantly between the two species across experiments, with 2 exceptions (Additional file 1: Table S1).

## Discussion

The susceptibility of *An. funestus* to *Plasmodium* infection remains relatively uncharacterised in laboratory conditions. The only two studies carried out to date were limited to the use of the rodent parasite *P. berghei* [22, 23]. This model organism is more amenable than *P. falciparum* and has been instrumental in malaria research studies leading to important discoveries on mechanisms of parasite killing in *Anopheles* vectors [12–14, 24, 38]. However, parallel analyses of *An. gambiae* transcriptional immune responses to *P. berghei* and *P. falciparum* has revealed substantial differences [14] pointing out the necessity to carry out such studies on the human malarial transmission system. Here, we report the first experimental infections of F1 progeny of field caught *An. funestus* with its natural human parasite *P. falciparum*.

The study of mosquito susceptibility requires that the technique of infection be able to produce efficient and robust infections in mosquito vectors. In the current study, we tested the use of artificial glass-parafilm membrane system to feed *An. funestus* mosquitoes with infected blood from gametocyte carriers. This method is clearly preferable to direct feeding assays for ethical reasons, including avoiding the risk of infection of study participants with cryptic pathogens that may be transmitted when using wild-collected mosquitoes. Besides it also offers the possibility to replace the donor’s serum with a non-immune AB one to avoid transmission reducing activity of serum components [39].

Nonetheless, the membrane feeding assays present two important weaknesses which could impact the outcome of infection experiments. First, an inappropriate blood handling procedure can result in decline or loss of gametocyte infectivity [40]. Secondly, the number of mosquitoes taking a blood meal could be reduced, especially when using newly collected field mosquito samples, which are not yet adapted to artificial feeding in laboratory conditions [41, 42]. This would explain the differences in feeding rate observed in this study between the *An. coluzzii* Ngousso lab strain and the first generation wild *An. funestus*.

In this study, mosquitoes became infected in the majority (over 75 %) of experiments demonstrating that the parafilm/glass feeders system and the blood handling procedure used for *An. gambaie* (*s.l*.) are also suitable for *An. funestus* experimental infections. However, the feeding rate of F1females from wild *An. funestus* mosquitoes varied significantly across experiments and did not exceed 50 % in all cases. This was lower than those previously reported in *An. funestus* Fumoz laboratory strain (over 50 %) and in *An. gambiae* (51.9 %) [22]. The difference could more likely be due to the use of mice feeding system in the study of Lo & Coetzee [22] which better mimics the natural feeding condition of *Anopheles* mosquitoes and/or to a better adaptation of the Fumoz lab strain to feed in lab conditions. By contrast, our results are comparable to those of a similar study conducted in Senegal [42] in which the authors also used the parafilm/glass feeder system to infect wild *An. arabiensis* F1 progeny and reported low feeding rate (32.7 %) further confirming that freshly field collected mosquitoes are not well adapted to feed on artificial membrane as stated above. Nonetheless, low feeding rate of wild *Anopheles* strains could be compensated by increasing the number of mosquitoes for infection experiments and this can now be easily achieved for *An. funestus*, since collection and rearing of this mosquito species have been significantly improved [15].

The infection parameters (mean prevalence of infection 25.66; median oocyst number 12.5, mean oocyst load 7.02) recorded in this study were higher than that reported in a similar one using the *An. funestus* insecticide resistant (FUMOZ) and susceptible (FANG) lab strains and *P. berghei* parasite (mean prevalence of infection 20 %) [22]. Conversely, wild *An. funestus* genotypes from Mali (West Africa) exhibited significantly higher prevalence of infection (62–97 %) when infected with *P. berghei* [23]. The variations in prevalence of infection between the three studies could be explained by three main factors which have been shown to significantly predict the proportion of mosquitoes that become infected after taking an infectious blood meal: (i) the difference in gametocyte densities in blood used for experimental infections [43]; (ii) the difference in gametocyte sex ratio in blood [44]; and more likely (iii) the difference of infectivity of *Plasmodium* species used [45]. However, it is noteworthy that *An. funestus* is a vector of *P. falciparum* and do not naturally interact with *P. berghei*. Therefore, by using natural isolate of the human malaria parasite *P. falciparum*, the present study gives the more realistic picture of its vector competence.

*Anopheles funestus* displayed high and comparable levels of susceptibility to *P. falciparum* infection in comparison to *An. coluzzii* as measured by the percentage of experiments showing infected mosquitoes, prevalence of infection and oocyst number in midguts. Our observations reinforce the view that both species are readily infected with *P. falciparum*, the most common and dangerous malaria parasite in sub-Saharan Africa, which may have had a greater ability to develop defense strategies against the mosquito immune system due to their long period of co-evolution [40].

Although our primary objective was not to investigate the kinetics of *P. falciparum* development in *An. funestus*, the fact that oocysts were observed in midguts after dissection of mosquitoes at day 7 gives preliminary indication that the parasite develops at similar rates to those in *An. gambiae* [46]. Further investigations with dissection of mosquitoes at later time points at which sporozoites should be observed in salivary glands are needed.

## Conclusion

This study reported high levels of susceptibility of wild *An. funestus* progeny to *P. falciparum* and since this mosquito is widespread throughout Africa, malaria vector control research and implementation need to seriously address this vector species too. Our results also demonstrate the applicability of the parafilm/glass feeding assays for *An. funestus* experimental infections with its natural parasite *P. falciparum*. This open new avenues toward further investigations of *An. funestus*-*Plasmodium* interactions in natural system, taking advantage of genomic tools now available.

## Abbreviations

ELISA, enzyme-linked immunosorbent assay; IRS, indoor residual house spraying; LLINs, long-lasting insecticide-treated nets; OCEAC, Organisation de Coordination pour la lutte contre les Endémies en Afrique Centrale

## Additional file

Additional file 1: Table S1. Infection parameters in *An. funestus* and *An. coluzzii* in nine parallel experiments. For each experiment, median oocyst number followed by the same letter are not significantly different; gametocyte densities correspond to the number of gamaetocytes per microliter of blood. (DOCX 16 kb)
