# Supplementary material for: High susceptibility of wild Anopheles funestus to infection with natural Plasmodium falciparum gametocytes using membrane feeding assays
Source: Parasit Vectors. 2016 Jun 14;9:341. doi: 10.1186/s13071-016-1626-y (PMC4908716; doi:10.1186/s13071-016-1626-y)
Supplement: Additional file 1: Table S1. — Infection parameters in An. funestus and An. coluzzii in nine parallel experiments. For each experiment, median oocyst number followed by the same letter are not significantly different; gametocyte densities correspond to the number of gamaetocytes per microliter of blood. (DOCX 16 kb) [file 13071_2016_1626_MOESM1_ESM.docx]

**Additional file 1: Table S1**  Infection parameters in *An. funestus* and *An. coluzzii* in nine parallel experiments. For each experiment, median oocyst number followed by the same letter are not significantly different; gametocyte densities correspond to the number of gamaetocytes per microliter of blood

| **Experiment**  **number** | **Species** | **Gametocyte density** | **Feeding rate (%)** | **Dissected** | **Infected** | **Infection**  **rate**  **(%)** | **Oocyst count** | **Oocyst**  **(Min-Max)** | **Median oocyst number** |
| --- | --- | --- | --- | --- | --- | --- | --- | --- | --- |
| N°1 | *An. funestus* | - | 24.52 | 32 | 11 | 34.38 | 45 | 1-15 | 2.5 ^a^ |
|  | *An. coluzzii* | - | 71.69 | 21 | 14 | 66.66 | 105 | 1-22 | 5 ^b^ |
|  |  |  |  |  |  |  |  |  |  |
| N°2 | *An. funestus* | - | 19.10 | 36 | 22 | 61.11 | 162 | 1-18 | 7 ^c^ |
|  | *An. coluzzii* | - | 61.49 | 57 | 23 | 40.35 | 57 | 1-9 | 3 ^d^ |
|  |  |  |  |  |  |  |  |  |  |
| N°4 | *An. funestus* | - | 25.25 | 32 | 2 | 6.25 | 04 | 1-3 | 1 ^e^ |
|  | *An. coluzzii* | - | 89.92 | 83 | 16 | 19.28 | 35 | 1-5 | 2.5 ^e^ |
|  |  |  |  |  |  |  |  |  |  |
| N°5 | *An. funestus* | - | 23.78 | 39 | 6 | 15.38 | 9 | 1-4 | 1 ^f^ |
|  | *An. coluzzii* | - | 55.81 | 73 | 5 | 6.85 | 6 | 1-2 | 1 ^f^ |
|  |  |  |  |  |  |  |  |  |  |
| N°6 | *An. funestus* | - | 46.53 | 24 | 3 | 12.5 | 04 | 1-2 | 1 ^g^ |
|  | *An. coluzzii* | - | 89.09 | 102 | 19 | 18.63 | 36 | 1-6 | 2.5 ^g^ |
|  |  |  |  |  |  |  |  |  |  |
| N°9 | *An. funestus* | 96 | 31.38 | 59 | 42 | 79.19 | 241 | 1-16 | 8 ^h^ |
|  | *An. coluzzii* |  | 76.24 | 113 | 84 | 74.34 | 505 | 1­-18 | 8 ^h^ |
|  |  |  |  |  |  |  |  |  |  |
| N°10 | *An. funestus* | 32 | 19.19 | 43 | 14 | 32.56 | 20 | 1-2 | 1 ^i^ |
|  | *An. coluzzii* |  | 86.44 | 21 | 4 | 19.05 | 04 | 1-1 | 0.5 ^i^ |
|  |  |  |  |  |  |  |  |  |  |
| N°12 | *An. funestus* | 880 | 18.37 | 9 | 9 | 100 | 539 | 1-139 | 60 ^j^ |
|  | *An. coluzzii* |  | 89.09 | 40 | 39 | 97.5 | 3878 | 2-351 | 90 ^j^ |
|  |  |  |  |  |  |  |  |  |  |
| N°13 | *An. funestus* | 80 | 31.92 | 105 | 37 | 35.24 | 96 | 1-8 | 4 ^k^ |
|  | *An. coluzzii* |  | 86.78 | 16 | 6 | 37.5 | 12 | 1-3 | 1.5 ^k^ |
|  |  |  |  |  |  |  |  |  |  |
| **All** | ***An. funestus*** |  | **29.23** | **379** | **146** | **38.52** | **1122** | **1-139** | **12.5** |
|  | ***An. coluzzii*** |  | **83.20** | **526** | **210** | **39.92** | **4269** | **1-351** | **32.1** |
